# NIST Standards for Microanalysis and the Certification Process

**DOI:** 10.6028/jres.107.055

**Published:** 2002-12-01

**Authors:** R. B. Marinenko

**Affiliations:** National Institute of Standards and Technology, Gaithersburg, MD 10899-8371

**Keywords:** certification, electron microprobe, microanalysis standards, microhomogeneity, microheterogeneity, standard reference materials for microanalysis

## Abstract

The National Institute of Standards and Technology (NIST) has been involved in the development of standards for microanalysis since the middle of the 1960s. Certification of “traceable” standards that can be sold to other laboratories is time-consuming and costly, especially when the extent of microheterogeneity within each specimen becomes part of the uncertainty assigned to the certified values. The process of certification of microanalysis standards and the improvements that have facilitated the process with the development of automation and computerization are reviewed.

## 1. Introduction

With the availability of the first commercially produced electron microprobe instruments in the early 1960s, x-ray microanalysis became a significant analytical technique in the Analytical Chemistry Division at NIST (then called the National Bureau of Standards). The person responsible for the development of electron probe microanalysis at NBS was Kurt. F. J. Heinrich under whose leadership the Microanalysis Section made numerous contributions to improvements in instrumentation, to the determination of fundamental constants, to the development of matrix correction procedures, and to the development of standard reference materials for microanalysis. NBS had been involved in the development of metrological and analytical standards from its beginning, but microanalysis standards presented the additional requirement of determining the extent of heterogeneity of a research material on the micrometer scale. Only materials that exhibited minimum microheterogeneity could be certified as NBS SRMs for microanalysis.

## 2. Early SRMs

During the mid-1960s, SRMs being sold as bulk standards were also evaluated for use as microanalysis standards. Those that were found sufficiently homogeneous to be used as microanalysis standards were Cartridge Brass (SRM 478) and Low-Alloy Steels (SRMs 461 and 463) [1]. In the early 1970s several binary and ternary alloys were issued as SRMs for microanalysis—a W-20%Mo Alloy (SRM 480) [2], Fe-3Si (SRM 483) [3], the Au-Ag alloys (SRM 481) and Cu-Au Alloys (SRM 482) [4], the Fe-Cr-Ni alloy (SRM 479 and 479a) [5,6], and a group of four different steels, (SRMs 661–664). These materials were chosen in part because they were useful standards for quantitative microanalysis and because they were useful in the determination of basic x-ray parameters. In addition, these materials could be made with little heterogeneity both in the bulk material (from specimen to specimen) and on the micrometer scale (within each specimen). These materials were not readily available commercially and if so, they certainly would not have been analyzed on the micrometer scale or certified at that level for microhomogeneity. Often special preparation procedures were required, such as repeated annealing, to achieve the desired level of homogeneity in metal alloys. This could be done with small batches of materials (like a few hundred grams) from which numerous microanalysis standards could be obtained.

Table 1 is a list of NBS/NIST SRMs for microanalysis that were certified between 1965 and the present. Many are no longer in stock (gray background), although for some (darker gray background) there is more material for a reissue if needed, but not without a considerable amount of work. Those with the complete white background are still in stock.

## 3. The Certification Process and More Recent SRMs

The question often asked is, “Why doesn’t NIST provide more microanalysis standards?” There are several reasons. Firstly, most pure elements and many stoichiometric compounds are available commercially, and many naturally occurring minerals are available. These can be easily purchased and subsequently evaluated by the user for microheterogeneity. In addition, there are a few commercial suppliers who purchase these commercially available materials, mount, polish, and evaluate them for resale as prepared microanalysis standards. NIST does not compete with such providers. Secondly, the fabrication and evaluation of research materials for certification as microanalysis standards is expensive and time-consuming. Details of the process will be described later. For these reasons, NBS/NIST scientists have concentrated their efforts on the development of more complex materials that were not available commercially and that might be more useful in quantitative electron probe microanalysis (EPMA), i.e., such as those that could be used in testing matrix correction procedures or in determining basic parameters.

During the latter part of the 1970s, glasses became popular as standards, throughout the microanalysis community. Because they are vitreous solids, many types of glasses can be made homogeneous on the micrometer scale. In addition, trace to minor amounts of elements can be added to glasses during the manufacturing process without changing the microhomogeneity. This fact provides the possibility of preparing standards with complex compositions. There are several limitations, though, with the use of glasses as standards. Not all oxides or phosphates or combination of oxides or phosphates readily form glasses, therefore limiting the number of glass matrices that can be produced. Also, not all oxides or salts can be introduced into a glass at all concentrations without creating some microheterogeneity. And most importantly, glasses, especially those composed of only low atomic number elements, are more susceptible to electron beam damage than are metals, therefore requiring special attention to electron beam sampling procedures.

Several glasses were certified as NBS or NIST SRMs. These include the *Glasses for Mineral Analysis* (SRM 470) [7], K-411 and K-412, composed of the oxides of Mg, Al, Si, Ca, and Fe. A second group of 15 glasses were in part certified as *Glasses for Microanalysis* (SRMs 1871–1875) [8]. Five different glass matrices were used for each SRM. In each SRM were three glasses, one of the glass matrix only and two with the same matrix but each containing several different oxides in concentrations of 1.0 mass fraction or less. Glass fibres of some of these glasses were also sold as NBS Research Materials. More recently, glass microspheres made from K-411, above, were issued as SRM 2066 [9]. K-411 was also used in the preparation of SRM 2063a, a glass film on a Cu grid that was issued as an AEM (analytical electron microscope) standard [10].

There are several misconceptions in the microanalysis community about NIST and SRMs for microanalysis. First, there is a belief, especially among those who are just entering the microanalysis field, that NIST provides all microanalysis standards, that NIST will have available whatever they need, and that NIST can provide a complete set of “traceable to NIST” microanalysis standards. This, of course, is not the case for reasons cited above. Second, there is also the belief that any NIST SRM can be used for microanalysis. This is false since most NIST SRMs are for use in bulk analyses and have not been tested for microheterogeneity. Third, the certified values are valid regardless of how the SRM is prepared for analysis. When certified by NIST, much care must be taken in mounting and polishing these materials for microanalysis, whether a standard or an unknown material. In some cases there are specific instructions on how to prepare an SRM for use as a microanalysis standard.

As previously mentioned, the certification of any SRM, whether for microanalysis or bulk analysis, is a time-consuming and therefore an expensive process. Many people contribute to the process—microanalysis scientists, members of the Standard Reference Materials Program (SRMP), statisticians, materials scientists, and, in some cases, others from inside or outside NIST. Therefore, the usefulness of the standard must be carefully evaluated before work is begun. In the microanalysis community there are a limited number of laboratories, therefore there may be some difficulty in selling enough SRMs to justify the investment in certification. Several questions must be asked. They are as follows:

Would it be a useful qualitative and quantitative standard for microanalysis?
Would it be useful for improving the understanding and/or determination of basic parameters?
Would it be useful for quality control?
Is this a material that is not readily available from commercial sources?
What is the sales potential of the material if it is certified?
How important would this material be for the commercial community if it were certified for microanalysis?

Once the work is justified, the research material must be fabricated if there is not already a source for it. In the past, some of the SRMs cited above were produced at NIST, such as the Fe-Cr-Ni alloy (SRM 479 and SRM 479a), as well as all of the glasses and the glass microspheres. Some were produced commercially, such as W-20%Mo Alloy (SRM 480) and the Au-Ag, Au-Cu alloys (SRMs 481 and 482). Of course, enough must be produced for distribution to potential purchasers. Initial evaluation would normally include bulk physical examination for such characteristics as clarity in the glasses and physical robustness to the environment as well as microscopic examination to evaluate the extent of voids, inclusions, and multiple phases. EPMA evaluation of the research material would include studies of backscatter and secondary electron images, qualitative and quantitative analyses, and testing of the within and between specimen heterogeneity. If the material were to fail the tests, i.e., if it appeared to be outside of the acceptable limits of micro- and macro- heterogeneity, it would be reprocessed, either refabricated or reannealed as occurred in the fabrication of the Au-Cu alloys [4]. Some SRMs, such as the asbestos SRMs, were naturally occurring, so a fabrication process was not necessary, but a rigorous sample preparation and evaluation testing procedure was necessary.

Usually, at this stage of the process when the material has been found satisfactory for SRM certification, funding must be acquired from SRMP to continue the work that may or may not have begun under some initial SRMP funding. If additional funds are not obtained, work would be delayed or terminated on the project.

If continued, a sampling strategy for heterogeneity testing is designed with the help of the NIST statisticians. Heterogeneity testing is an extremely important part of the process of certifying an SRM as a microanalysis reference standard. Unless individually assigned certified composition and uncertainty values, the specimen sold to the electron microprobe laboratory must have the same certified composition as the rest of the SRM batch and the same uncertainty in the extent of heterogeneity for all certified elements. To assure that this is true, the research material must be thoroughly evaluated for microheterogeneity within specimens and for heterogeneity between specimens. In the early days before instruments were automated, sampling strategies used analysis of random points, traverses such as chords and diameters, as well as random location samplings on the specimens [1–3]. When computers first became available to assist in the sampling and data analysis, two-dimensional arrays and periodic integrator traces of traverses facilitated the acquisition of data [4,11]. Repetition of the sampling procedure with more than one operator was routine to be sure that there was not a systematic uncertainty introduced in the analysis by false judgements of an operator. Today, with completely automated electron microprobes, the design of the sampling strategy depends on the number and size of the specimens and the time available to do the work. Each research material requires its own individual approach, but in most cases, when dealing with flat, polished specimens, traverses and possibly also array sampling, are included in the overall testing procedure. If the lot of samples is large, representative sampling is used. Such was the case when a glass bar was cut up into several hundred rods, 2 mm × 2 mm × 10 mm. Seven to ten were randomly selected from the batch, mounted, polished, and carbon-coated for EPMA. Each specimen was sampled on at least seven different random points with two or three readings taken from each point. For other materials, such as the TiAl alloy that is presently being evaluated, all 27 2.54 mm specimens that had been cut from the same rod were tested before three were cut up for quantitative bulk analysis and for microanalysis standards. Since these TiAl specimens were large enough, x-ray fluorescence analysis was also used to evaluate between specimen heterogeneity. A very large specimen, such as a 7.5 cm wafer, can be evaluated by dividing it into sectors and treating each sector as a different specimen. This can be later cut up for distribution to other laboratories. Whether or not the testing is done under automation or not, the purpose is to efficiently obtain a good statistical evaluation of the within-specimen and between-specimen heterogeneity.

The sampling strategy discussed above is called a “nested design” and is described in more detail elsewhere [12,13]. The purpose is to obtain data that can be separated systematically into the components of variance for the within-specimen, between-specimen, and experimental uncertainties [14]. From known compositions of each element in the research material and the spectral background for each element that is subtracted from the total number of counts observed for each element, each variance can be converted from x-ray counts to a mass fraction value. For each element, the square root of the sum of the three variances is then the uncertainty (66.7 %) in the research material attributed to the heterogeneity. With the aid of statisticians, this uncertainty is combined with the uncertainty in the bulk quantitative analysis of the material to obtain the uncertainty in the certified value for each element [15].

Today, as in the past, heterogeneity testing at NIST has been done with wavelength dispersive spectrometers (WDS) set up at the optimum conditions that would be used for a good quantitative analysis. The objective is to select the elemental characteristic x-ray line, the crystal, the excitation potential, and the current to obtain the best count rate possible without exceeding the maximum count rate limit recommended for the x-ray detector and without damaging the specimen with the electron beam. Energy dispersive spectrometers (EDS) can also be used, measuring peak integrals instead of the x-ray counts at the peak maximum as for WDS, but the acquisition time would be extended considerably. Excellent instrument stability (less than 1 %) over long time periods (10 h to 12 h) or a very accurate drift correction procedure would be needed. EDS was used for quantitative analysis of the K-411 Glass Microspheres and the Microanalysis Thin Film Standards, but these were not flat, polished research materials like the rest of the SRMs and were both certified by different procedures.

Although the research material is quantitatively analyzed with EPMA, more accurate techniques are used for determining the certified bulk composition values that are ultimately assigned to the SRM. EPMA results usually appear only as information values on the certificate. Of course there must be enough material (usually 3 g to 4 g) in the original lot of the research material to provide specimens for bulk analysis. The techniques often used for the most accurate results are ICP-MS, ICP-OES, and XRF. Classical gravimetry has also been used in the past. Quantitative analysis by more than one technique and/or laboratory is preferred. Results from techniques are weighted [15] and combined by the NIST statistician to obtain a certified composition; subsequently, the heterogeneity uncertainty is combined with the uncertainty in the quantitative analyses to obtain the uncertainty of each element for the certificate. To close off the certification process, a Report of Analysis must be written and an SRM Certificate must be prepared. The latter is done in coordination with SRMP.

## 4. Conclusion

This manuscript describes the process used at NIST for the certification of microanalysis SRMs. More detailed descriptions of the manufacturing process and analyses can be found in the cited references. Certificates, descriptions of the materials, and prices can be found on the web site http://www.nist.gov/srm/.

## Figures and Tables

**Table 1:** NIST standard reference materials for microanalysis

SRM no	Name	Form	Nominal composition (% Mass fract.)
461 & 463	Low Alloy Steel	Rods, ≅ 6 mm dia. ×10 cm long	Fe with plus 25 other elements at or near trace level concentrations
470	Mineral Glasses for Microanalysis	Slices (2 × 2 × 12) mm^3^	K-411, MgO,SiO_2_,CaO,FeO K-412, MgO,Al_2_O_3_SiO_2_,CaO,FeO
478	Cartridge Brass	Cube and Cylinder	Cu-73, Zn-27
479a	Fe-Cr-Ni Alloy	Wafer	Fe-71,Cr-18,Ni-11
480	Tungsten 20 % Molybdenum	Wafer	W-78,Mo-22
481	Gold-Silver Alloys	Six wires	Au 100;80;60;40;20;0 Ag 0;20;40;60;80;100
482	Gold-Copper Alloys	Six wires	Au 100;80;60;40;20;0 Cu 0;20;40;60;80;100
483	Iron–3 % Silicon	Small sheet	FE-97, SI-3
1871–1875	Glasses for Microanalysis (1872, 1873 in stock)	Slices (2 × 2 × 12) mm^3^	15 Compositions of Various Oxides
2063a	Microanalysis Thin Film Mg-Si-Ca-Fe	Film on 3 mm Cu grid	K-411 glass (MgO,SiO_2_,CaO,FeO) used to prepare film
2066	K-411 Glass Microspheres	50 mg of 1 μm to 40 μm diameter spheres	K-411 glass (MgO,SiO_2_,CaO,FeO) used to make spheres
661	AISI 4340 Steel	3.2 mm ×51 mm rods	Steels with the several additional elements in minor and trace amounts
662	AISI 84B17 Steel Cr-V		
663	Steel High-Carbon		
664	Steel (Modified)		
